# Prostate-Specific Antigen 5 Years following Stereotactic Body Radiation Therapy for Low- and Intermediate-Risk Prostate Cancer: An Ablative Procedure?

**DOI:** 10.3389/fonc.2017.00157

**Published:** 2017-07-24

**Authors:** Shaan Kataria, Harsha Koneru, Shan Guleria, Malika Danner, Marilyn Ayoob, Thomas Yung, Siyuan Lei, Brian T. Collins, Simeng Suy, John H. Lynch, Thomas Kole, Sean P. Collins

**Affiliations:** ^1^Department of Radiation Medicine, Georgetown University Hospital, Washington, DC, United States; ^2^Department of Urology, Georgetown University Hospital, Washington, DC, United States; ^3^Department of Radiation Oncology, Valley Hospital, Ridgewood, NJ, United States

**Keywords:** stereotactic body radiation therapy, prostate cancer, PSA bounce, PSA recurrence, PSA kinetics, biochemical relapse, PSA, ablation

## Abstract

**Background:**

Our previous work on early PSA kinetics following prostate stereotactic body radiation therapy (SBRT) demonstrated that an initial rapid and then slow PSA decline may result in very low PSA nadirs. This retrospective study sought to evaluate the PSA nadir 5 years following SBRT for low- and intermediate-risk prostate cancer (PCa).

**Methods:**

65 low- and 80 intermediate-risk PCa patients were treated definitively with SBRT to 35–37.5 Gy in 5 fractions at Georgetown University Hospital between January 2008 and October 2011. Patients who received androgen deprivation therapy were excluded from this study. Biochemical relapse was defined as a PSA rise >2 ng/ml above the nadir and analyzed using the Kaplan–Meier method. The PSA nadir was defined as the lowest PSA value prior to biochemical relapse or as the lowest value recorded during follow-up. Prostate ablation was defined as a PSA nadir <0.2 ng/ml. Univariate logistic regression analysis was used to evaluate relevant variables on the likelihood of achieving a PSA nadir <0.2 ng/ml.

**Results:**

The median age at the start of SBRT was 72 years. These patients had a median prostate volume of 36 cc with a median 25% of total cores involved. At a median follow-up of 5.6 years, 86 and 37% of patients achieved a PSA nadir ≤0.5 and <0.2 ng/ml, respectively. The median time to PSA nadir was 36 months. Two low and seven intermediate risk patients experienced a biochemical relapse. Regardless of the PSA outcome, the median PSA nadir for all patients was 0.2 ng/ml. The 5-year biochemical relapse free survival (bRFS) rate for low- and intermediate-risk patients was 98.5 and 95%, respectively. Initial PSA (*p* = 0.024) and a lower testosterone at the time of the PSA nadir (*p* = 0.049) were found to be significant predictors of achieving a PSA nadir <0.2 ng/ml.

**Conclusion:**

SBRT for low- and intermediate-risk PCa is a convenient treatment option with low PSA nadirs and a high rate of early bRFS. Fewer than 40% of patients, however, achieved an ablative PSA nadir. Thus, the role of further dose escalation is an area of active investigation.

## Introduction

Men with prostate cancer (PCa) treated with prostatectomy generally achieve an undetectable PSA (<0.1 ng/ml) within weeks to months (1). A detectable PSA is highly associated with future clinical progression. Not all patients, however, will progress to local or distant disease recurrence because an elevated PSA may also be due to residual benign prostate tissue (2). For this reason, the American Urological Association defines biochemical recurrence as a PSA ≥0.2 ng/ml (3). The value of ultrasensitive PSA tests (sensitivity of ≤0.01 ng/ml) in identifying early recurrences has yet to be shown (4, 5).

Conventionally fractionated external beam radiation therapy (EBRT) results in a slow PSA declines that then stabilizes. A PSA nadir to <0.5 ng/ml predicts for lower rates of biochemical failure (Phoenix definition: 2 ng/ml above nadir) and distant failure (6). PSA nadirs to undetectable levels are uncommon because this technique does not fully ablate normal prostate tissue. Any subsequent PSA rises following the nadir may be due to benign PSA bounces, local recurrence or distant metastatic disease. Benign PSA bounces are generally small (<1 ng/ml) and transient, resolving over months to years (7).

Following brachytherapy, PSA nadirs are typically lower than conventionally fractionated EBRT with 90% of patients reaching a nadir of <0.5 ng/ml at 5 years posttreatment and a median PSA of 0.1 ng/ml when measured with ultrasensitive PSA testing (8). In a study by Lo et al., if a patient’s PSA was ≤0.4 ng/ml at 4 years post implantation, he had a >99% chance of being free of disease at 8 years following treatment (9). The median PSA nadir for that study was 0.05 ng/ml, suggesting that LDR brachytherapy performed in such a manner was potentially ablative. Patients with a 5-year PSA value of <0.2 ng/ml have a 1–2% risk of developing biochemical failure within the next 5 years (10, 11).

Stereotactic body radiation therapy (SBRT) has emerged as a viable treatment option for many of the over 220,000 patients diagnosed with PCa in the United States each year (12). The American Society for Radiation Oncology defines SBRT as “an external beam radiation therapy method that very precisely delivers a high dose of radiation to an extracranial target” (13). Many terms have been used to describe SBRT including “stereotactic body ablative radiotherapy” and “extreme hypofractionation” (14, 15). In general, SBRT delivers 35–40 Gy in 4–5 fractions, resulting in an overall lower PSA nadir compared to those treated with intensity modulated radiation therapy; these patients also exhibit a more rapid PSA decline in the second and third years post-SBRT (16–20). Dose escalation with SBRT has not been shown to improve biochemical relapse free survival (bRFS) in intermediate risk patients but may lead to worse toxicity (21, 22). This study sought to evaluate the PSA nadir 5 years following SBRT for PCa.

## Materials and Methods

### Patient Selection

Patients eligible for study inclusion had histologically confirmed low- or intermediate-risk PCa treated with SBRT monotherapy per our institutional protocol and were followed for a minimum of 5 years. Patients who received androgen deprivation therapy were excluded from the study due to its known impact on the PSA nadir (23). Clinical stage was defined according to the seventh edition of the American Joint Committee on Cancer. Risk groups were defined using the D’Amico criteria. Approval from the MedStar Research Institute-Georgetown University Oncology Institutional Review Board (IRB) was obtained for retrospective review of data that were prospectively collected in our institutional database (IRB#: 2009-510). The requirement to obtain written informed consent from research participants was waived by the Committee.

### SBRT Treatment Planning and Delivery

Stereotactic body radiation therapy was delivered utilizing the CyberKnife robotic radiosurgical system (Accuray Inc., Sunnyvale, CA, USA). Required fiducial placement and computed tomography (CT)/magnetic resonance simulation procedures have been previously described (24). The clinical target volume (CTV) was defined as the prostatic capsule and proximal seminal vesicles identified on the pretreatment CT and/or MRI. The CTV to planning target volume (PTV) expansion was 5 mm in all directions except 3 mm posteriorly into the rectum. Fiducial-based tracking was utilized to account for interfraction and intrafraction prostate motion (25). Treatment planning and dose calculations were performed using Multiplan (Accuray Inc., Sunnyvale, CA, USA). Patients were treated to 35, 36.25, or 37.5 Gy delivered in 5 fractions to the PTV. Assuming an α/β ratio of 1.5, this prescription corresponds to a tumor equivalent dose in 2-Gy fractions of approximately 85–90 Gy. The prescription isodose line, however, was limited to ≥75% to restrict the maximum prostatic urethra dose to 133% of the prescription dose.

### Follow-up and Statistical Analysis

Prostate-specific antigen and total testosterone levels were obtained prior to treatment and posttreatment every 3 months for the first year, every 6 months for the following 2 years, and yearly thereafter. Evaluated treatment response parameters included time to PSA nadir and the nadir value per individual patient. In addition to PSA kinetics, outcome measures studied included biochemical relapse as defined by the Phoenix definition (e.g., nadir + 2 ng/ml) (23). In patients with a biochemical relapse, PSA nadir was defined as the lowest PSA prior to failure; in patients without a biochemical relapse, PSA nadir was defined as the lowest PSA recorded during follow-up. A benign PSA bounce was defined as a PSA rise of 0.2 ng/ml or greater that then returned to the previous PSA nadir or lower (7). Prostate ablation was defined as a PSA nadir <0.2 ng/ml (2). Univariate logistic regression analysis was used to evaluate relevant variables on the likelihood of achieving a PSA nadir <0.2 ng/ml.

## Results

From January 2008 to October 2011, 145 PCa patients were treated per our institutional SBRT monotherapy protocol (Table 1). By D’Amico classification, 65 patients were low risk, and 80 were intermediate risk. The median age at the start of SBRT was 72 years, and 40.7% of patients were non-Caucasian. The median prostate volume was 36 cc with a median 25% of cores involved. 86.9% of the patients were treated with 36.25 Gy in five 7.25 Gy fractions.

**Table 1:** Patient, tumor, and treatment characteristics.

	*n* = 145
**Age (years)**	
Mean	72
Median (range)	72 (53–90)
**Race**	
White	86 (59.3%)
Black	52 (35.9%)
Other	7 (4.8%)
**BMI**	
18.5–24.9	24 (16.6%)
25–29.9	63 (43.4%)
≥30	58 (40%)
**Risk group (D’Amico)**	
Low	65 (44.8%)
Intermediate	80 (55.2%)
**Clinical stage**	
T1c–T2a	128 (88.3%)
T2b	17 (11.7%)
**Gleason score**	
≤6	74 (51%)
7 (3 + 4)	49 (33.8%)
(4 + 3)	21 (14.5%)
**PSA (ng/ml)**	
Mean	6.54
Median	5.70 (1.8–18.6)
**Testosterone**	
Mean	357.29
Median	316 (126–1,149)
**Prostate volume (cc)**	
Mean	39.19
Median	36.05 (11.56–138.69)
**# of Cores involved**	
Mean	3
Median	3 (1–10)
**% of Total cores positive**	
Mean	30.2
Median	25 (3–100)
**Maximum % of a single involved core**	
Mean	37.79
Median	30 (2–95)
**Treatment dose (Gy)**	
35	15 (10.3%)
36.25	126 (86.9%)
37.5	74 (2.8%)

At a median follow-up of 5.6 years (range 5–7.4 years), 84 and 37% of patients achieved a nadir ≤0.5 and <0.2 ng/ml, respectively, and the median time to PSA nadir was 36 months. There were nine biochemical relapses, occurring in two low risk and seven intermediate-risk patients; the median time to relapse was 4.1 years. Regardless of the PSA outcome, the median PSA nadir for all patients was 0.2 ng/ml (Table 2). Benign PSA bounces were identified in 58 patients (40%), and four patients experienced a PSA bounce of greater than 2 ng/ml. The percentage of patients without a biochemical relapse reaching a given PSA nadir during follow-up is described in Table 3. There was little difference between the pretreatment testosterone level and the testosterone level measured at the PSA nadir.

**Table 2:** PSA outcomes at a median follow-up of 5.6 years.

	All (*n* = 145)
# of Biochemical failures	9
Median months to biochemical failure	50 (34–81)
Median PSA nadir (ng/ml)	0.2 (0.02–2.1)
Median months to nadir	36 (3–84)
PSA nadir ≤0.5	122 (84%)
PSA nadir <0.2	54 (37%)
Median testosterone at PSA nadir	305 (73–1,423)

**Table 3:** The percentage of patients with biochemical RFS reaching a given PSA in the years following stereotactic body radiation therapy.

PSA (ng/ml)	1 (year)	2	3	4	5
≤1	61%	90%	95%	96%	93%
≤0.5	27%	63%	72%	75%	73%
≤0.2	6%	20%	37%	43%	45%
≤0.1	2%	7%	17%	24%	23%
<0.1	0%	0%	0%	1%	7%
*N*	125	126	130	121	128

**Figure 1:**
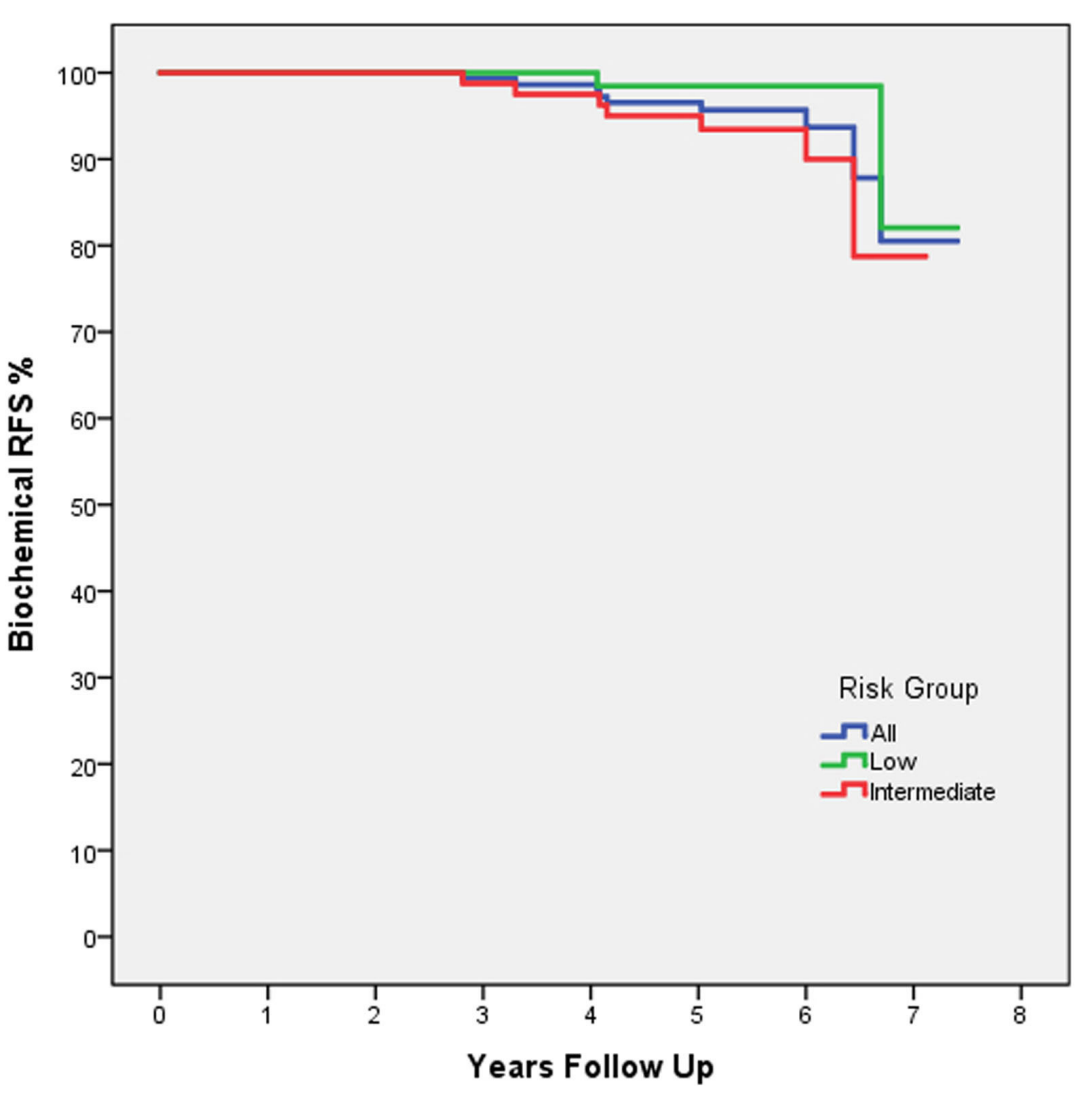
Biochemical RFS by D’Amico risk group: all patients (blue), low risk (green), and intermediate risk (red). The dashed line indicates the 5-year follow-up time point.

**Figure 2:**
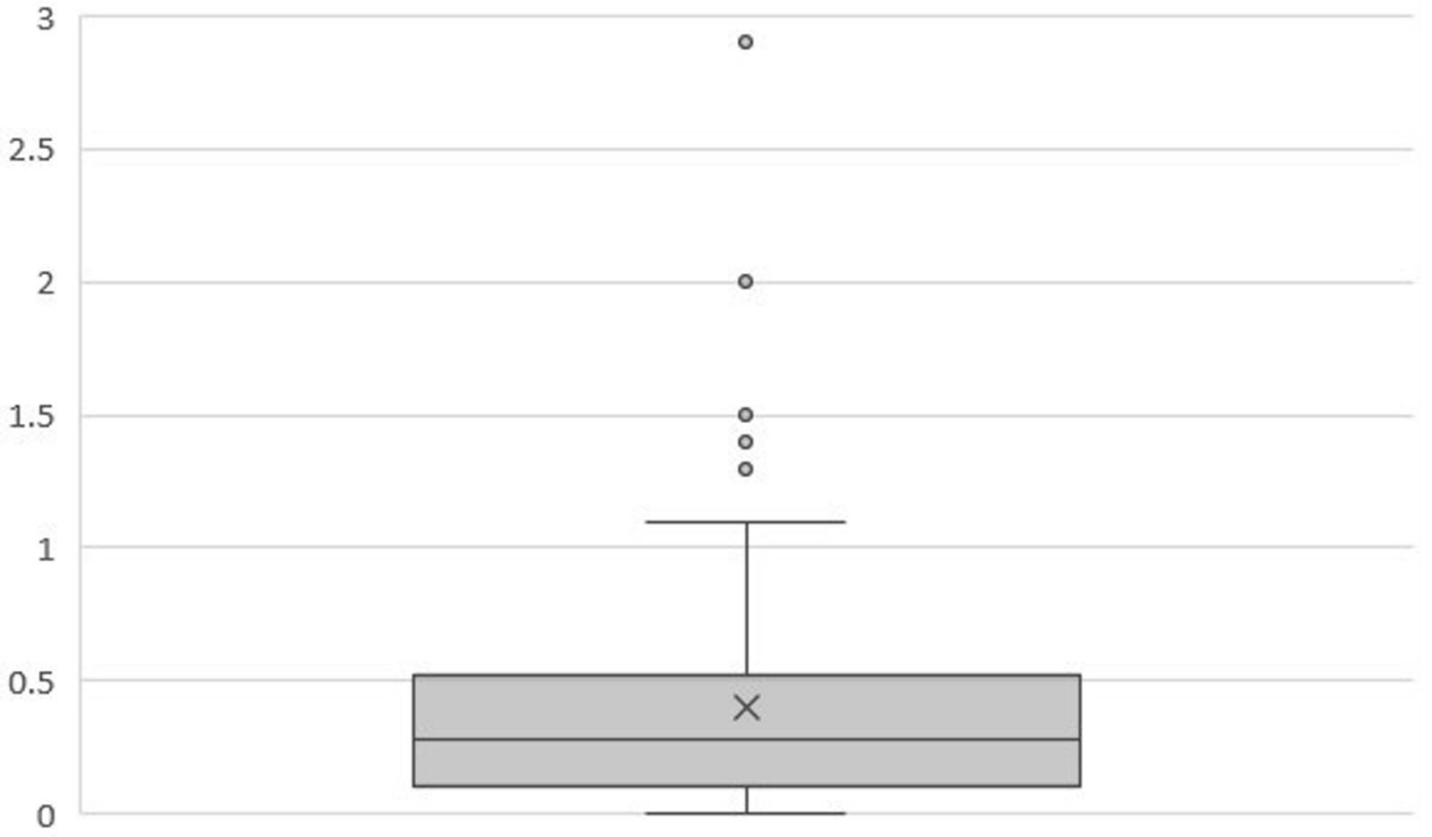
Box plot illustrating the distribution of PSA values (ng/ml) at 5 years in patients without biochemical relapse (*n* = 128). Mean = 0.40. Median = 0.28. *Q*1 = 0.1. *Q*3 = 0.52.

At 5 years posttreatment, the median pretreatment PSA of 5.7 ng/ml (range, 1.8–18.6 ng/ml) declined to a median of 0.3 ng/ml (range, 0–53 ng/ml). There was no statistically significant difference between the 5-year bRFS rate for low- (98.5%) and intermediate-risk (95.5%) patients (Figure 1). The distribution of PSA values at 5 years in patients without biochemical relapse is illustrated in Figure 2.

A PSA nadir of <0.2 ng/ml was selected to estimate the likelihood of achieving long-term bRFS. Specific clinical features such as age, prostate volume, T-stage, Gleason score, baseline PSA, pretreatment testosterone, testosterone at the time of PSA nadir, time to PSA nadir, and the percentage of cores involved were evaluated as predictors for achieving a PSA <0.2 ng/ml. Utilizing univariate logistic regression analysis, only a lower baseline PSA and a lower testosterone level at the time of the PSA nadir were found to be significant predictors of achieving a PSA <0.2 ng/ml (Table 4).

**Table 4:** Univariate logistic regression.

Univariate logistic regression			
Variable	OR	95% CI	*p*-Value
Age (years)	0.997	(0.948–1.049)	0.906
Prostate volume (cm^3^)	0.981	(0.959–1.004)	0.1
T-stage			
T1c–T2a	1		
T2b–T2c	1.137	(0.306–2.530)	0.812
Gleason score			
≤6			
7	1.134	(0.579–2.218)	0.714
PSA, baseline (ng/ml)	0.856	(0.748–0.980)	**0.024**
Testosterone, baseline (ng/dl)	1	(0.998–1.002)	0.862
Testosterone, nadir (ng/dl)	0.997	(0.993–1.000)	**0.049**
Time to PSA nadir (months)	1.014	(0.997–1.033)	0.116
% Positive cores involved	1.013	(0.997–1.029)	0.11

*Predictors of achieving a PSA nadir <0.2 ng/ml*.

*p values in bold are statistically significant*.

## Discussion

Conventionally fractionated EBRT is designed to safely eradicate cancerous cells while minimizing the dose to surrounding normal tissues and reducing the risk of unnecessary toxicity. Utilization of this technique, however, may not be adequate to eradicate all cancer cells with the most common site of recurrence being within the prostate (26). Ablation is the destruction of cancerous cells and/or normal tissue by large radiation doses via vascular mediated necrosis (27). Unfortunately, delivering SBRT with ablative doses of radiation to the entire prostate (>40 Gy) have led to high rates of >Grade 3 toxicity (21). Doses of 35–40 Gy in 4–5 fractions have been found to be safe while achieving PSA nadirs lower than conventionally fractionated EBRT (17, 28). It remains unclear if these doses are adequate for long-term (>20 years) local control.

Recently published data indicate that fewer than 20% of low risk PCa patients under the age of 65 years at diagnosis are treated with conventionally fractionated EBRT, while nearly 50% of patients are treated with radical prostatectomy (29). This trend may be due to the fact that many physicians believe that young men with PCa are at a higher risk of treatment failure following primary conventionally fractionated EBRT due to their predicted long life expectancy and the non-ablative nature of this radiotherapy technique (30). Not all men are ideal candidates for surgery and conventionally fractionated EBRT requires >8 weeks of daily treatment. Furthermore, both prostatectomy and conventionally fractionated EBRT result in similar long-term declines in functional outcomes (31).

Brachytherapy is a well-established alternative to surgery for these men due to the achievement of very low PSA nadirs when administered following strict guidelines (32). Patients treated with low-dose brachytherapy with a biologic effective dose (BED) >180 Gy resulted in 10-year freedom from PSA failure rates of >90% (33). Not all patients, however, are ideal candidates for brachytherapy (i.e., large prostate, prior TURP or high IPSS) (34) and are looking for a convenient alternative. SBRT delivered to 35–37.25 Gy in 5 fractions provides a BED of >198 Gy resulting in greater coverage of the CTV and a more conformal and homogeneic dose distribution compared to brachytherapy. Our 5-year bRFS for low- (98.5%) and intermediate-risk (95.5%) patients are comparable to those reported for high dose conventionally fractionated radiation therapy and low-dose brachytherapy (35–37). Further follow-up of PCa patients treated with SBRT will be required to confirm long-term cancer control suggested by its low PSA nadir.

This study has several identifiable limitations. First, PSAs may continue to decline for years after prostate radiotherapy, and 5 years of follow-up may be inadequate to assess the ultimate PSA nadir for an individual patient. Second, the PSA nadir might not be an ideal indicator of long-term cancer control due to its dependence on the pretreatment PSA and the testosterone level at the time of PSA nadir. Finally, most patients were followed utilizing standard PSA tests with a sensitivity of 0.1 ng/ml. A standard approach was not utilized when ultrasensitive PSA tests were performed that resulted in values <0.1 ng/ml. When the ultrasensitive test was employed, however, several patients achieved nadirs as low as 0.05 ng/ml. Therefore, it is likely that the mean PSA nadir would have been lower if the ultrasensitive tests were routinely employed.

## Conclusion

Stereotactic body radiation therapy for clinically localized low- and intermediate-risk PCa is a convenient treatment option with low PSA nadirs suggestive of high rates of early bRFS. In this study, although fewer than 40% of patients achieved an ablative PSA nadir, high rates of bRFS have been observed. Further follow-up of PCa patients treated with SBRT monotherapy is required to determine the long-term outcomes of this technique. The role of further dose escalation is an area of active investigation.

## Author Contributions

SK, HK, SG, SS, and TK contributed in data collection and analysis. MD contributed as a research coordinator. SL, MA, TY, BC, JL, TK, and SC contributed in treatment planning, the delivery of stereotactic body radiation therapy, and in patient care pre- and posttreatment.

## Conflict of Interest Statement

SC and BC serve as clinical consultants to Accuray Inc. The Department of Radiation Medicine at Georgetown University Hospital receives a grant from Accuray to support a research coordinator. The other authors declare that they have no competing interests.

## Abbreviations

ADT, androgen deprivation therapy; CT, computed tomography; CTV, clinical target volume; EQD2, equivalent dose in 2-Gy fractions; IMRT, intensity modulated radiation therapy; IRB, institutional review board; PTV, planning target volume; MR, magnetic resonance; SBRT, stereotactic body radiation therapy.
